# Electrophysiological asymmetry in vincristine-exposed children with acute lymphoblastic leukemia: Evidence from bilateral nerve conduction studies

**DOI:** 10.1371/journal.pone.0352440

**Published:** 2026-06-29

**Authors:** Filip Jevic, Ross Andel, Josef Kraus, Alena Kobesova

**Affiliations:** 1 Department of Rehabilitation and Sports Medicine, Second Faculty of Medicine, Charles University and University Hospital Motol and Homolka, Prague, Czech Republic; 2 Charles University, Third Faculty of Medicine, Prague, Czech Republic; 3 Edson College of Nursing and Health Innovation, Arizona State University, Phoenix, Arizona, United States of America; 4 Department of Neurology, Second Faculty of Medicine, Charles University and University Hospital Motol and Homolka, Prague, Czech Republic; 5 Department of Pediatric Neurology, Second Faculty of Medicine, Charles University and University Hospital Motol and Homolka, Prague, Czech Republic; Tokai University School of Medicine, JAPAN

## Abstract

Vincristine-induced peripheral neuropathy (VIPN) is a frequent complication of therapy for acute lymphoblastic leukemia (ALL) in children. Beyond acute toxicity, VIPN may affect motor development, balance, and quality of life in survivors. Although often assumed to be symmetric, the extent of electrophysiological asymmetry has not been systematically evaluated. This study aimed to quantify side-to-side differences in vincristine-exposed children using bilateral nerve conduction studies (NCS). Forty-seven bilateral NCS assessments were performed in 47 children with ALL (32 post-treatment survivors and 15 on active therapy). Distal latencies, amplitudes, and conduction velocities were compared between sides using intraclass correlation coefficients (ICC), Cohen’s kappa, and McNemar’s test at three hierarchical levels: individual parameters, nerve-level classifications, and limb-level neuropathy. Motor nerve parameters, particularly compound muscle action potential amplitude and conduction velocity, showed only fair to moderate inter-side agreement (ICC range 0.28–0.64), with the lowest concordance observed in the peroneal and ulnar nerves. In contrast, sensory latencies demonstrated excellent symmetry (ICC > 0.90). Whole-nerve classifications for motor nerves revealed fair to moderate agreement (kappa 0.38–0.49), while NCS-defined polyneuropathy classification showed substantial agreement (kappa = 0.66). Furthermore, marked amplitude asymmetry (side-to-side ratio < 0.5) was observed in 40.4% of peroneal nerves. These results suggest that electrophysiological asymmetry is a frequent feature of pediatric VIPN and may have clinical relevance. Recognition of asymmetry does not fully align with the traditional view of VIPN as a symmetric neuropathy and supports consideration of bilateral NCS in clinical and research settings. Improved awareness of asymmetry may help improve diagnostic accuracy and inform the development of future assessment criteria. Because asymmetry could contribute to postural imbalance, gait deviations, and reduced functional capacity, its detection may be relevant for rehabilitation planning and for guiding future studies on long-term motor outcomes in this pediatric population.

## 1 Introduction

Acute lymphoblastic leukemia (ALL) is the most common pediatric malignancy, with 5-year survival exceeding 90 percent in high-income countries [1]. As outcomes improve, treatment-related complications like vincristine-induced peripheral neuropathy (VIPN) are gaining focus [2,3]. VIPN is among the most frequent chemotherapy side effects in children with ALL and may persist throughout treatment [4], with potential consequences for motor development and daily functioning in survivors [5]

VIPN incidence ranges from 2.8 to 100 percent, depending on risk factors and assessment methods [6,7]. Clinical tools such as the Common Terminology Criteria for Adverse Events (CTCAE) [8], Balis scale [9], Pediatric Neuropathic Pain Scale [10], the pediatric-modified Total Neuropathy Score (ped-mTNS) [11], and the Total Neuropathy Score – Pediatric Vincristine are commonly used [12]. These scales, however, provide global severity scores and do not capture side-to-side differences, potentially overlooking asymmetry. Nerve conduction studies (NCS) remain the ‘gold standard’ for early, objective VIPN detection [13], offering greater sensitivity [9] and detailed neuropathy characterization [4].

Reported VIPN patterns can be described along three dimensions. In terms of fiber involvement, neuropathy is usually motor-predominant [14] or sensorimotor [7], with no consistently reported cases of pure sensory electrophysiological patterns. In terms of pathophysiology, most studies indicate axonal injury [15–18], occasionally with mixed axonal–demyelinating features [19]. Finally, in terms of distribution, VIPN is most often length-dependent [20–22], but non-length-dependent patterns have also been observed [14], particularly in younger children [23]. Despite this variability, most studies including recent comprehensive reviews characterize VIPN as a strictly symmetric, distal neuropathy [20–22,24,25].

This assumption has rarely been systematically tested. Some studies used bilateral NCS but assessed only one nerve [24] or lacked quantitative comparisons [25]. Others used unspecified NCS protocols [21] or assumed symmetry without testing it [22], limiting their reliability.

Nonetheless, several findings suggest asymmetry. Clinical asymmetry has been reported in vincristine-treated children [26], and bilateral NCS revealed side-to-side differences in peroneal and sural nerves [14]. One study described an asymmetric, non-length-dependent pattern consistent with mononeuritis multiplex [27]. These findings challenge the symmetry assumption. To date, no study has quantitatively assessed side-to-side differences across multiple nerves in a homogeneous pediatric ALL cohort. Recent reviews also highlight inconsistent assessment protocols and limited NCS use in VIPN [28].

This study aimed to systematically assess electrophysiological asymmetry in vincristine-exposed children with ALL using bilateral NCS. We hypothesized that VIPN may not consistently follow a symmetric pattern. We also examined whether abnormalities align with features of distal symmetric polyneuropathy (DSP) described in adults [29], using this framework for reference only, as no standard pediatric DSP criteria exist. Through multi-level analysis of bilateral NCS data across several nerves, we investigated whether asymmetry is a consistent feature of VIPN and may have clinical relevance. Clarifying asymmetry could help improve diagnostic accuracy and may provide a basis for future rehabilitation research on secondary postural problems and long-term motor outcomes.

## 2 Materials and methods

### 2.1 Participants

This single-center observational study utilized a cross-sectional design to assess electrophysiological asymmetry in nerve conduction parameters between left and right sides (within-subject comparison) in pediatric patients with acute lymphoblastic leukemia (ALL). The sample included two clinical subgroups: (1) post-treatment survivors and (2) patients currently undergoing active chemotherapy. To ensure strict statistical independence of observations, the analysis included only a single NCS assessment per participant. For post-treatment survivors, their single cross-sectional assessment was used. For patients in active chemotherapy, who were routinely assessed twice during their treatment protocol, only the second assessment (conducted at Week 21) was included in this analysis, as this time point represents a more established state of cumulative neurotoxicity.

Participants were recruited between November 23, 2020 and May 31, 2024 from the Department of Paediatric Haematology and Oncology, Second Faculty of Medicine, Charles University in Prague and Motol University Hospital. Eligible participants were identified through the oncology registry. The post-treatment group was enrolled and assessed between November 23, 2020, and December 16, 2021. The treatment group was assessed between January 10, 2022 and May 31, 2024. Identifiable participant data were accessible to the research team; however, all data were anonymized prior to analysis.

Inclusion criteria for both groups were: (1) ALL as the first primary malignancy and (2) age between 4 and 19 years at assessment. Exclusion criteria were: (1) ALL relapse, (2) neurologic, genetic, or developmental disorders diagnosed prior to ALL, and (3) cranial radiotherapy during treatment due to potential impact on neurodevelopment [30]. Post-treatment survivors were treated under the AIEOP BFM ALL 2009 protocol [31], with treatment completed 1 month to 4 years prior to enrollment (mean 2.6 years, SD 1.1, range 1.1 to 5.0). Patients in treatment followed the AIEOP BFM ALL 2017 protocol and underwent NCS assessments during weeks 8 and 21 of therapy.

**Fig 1:**
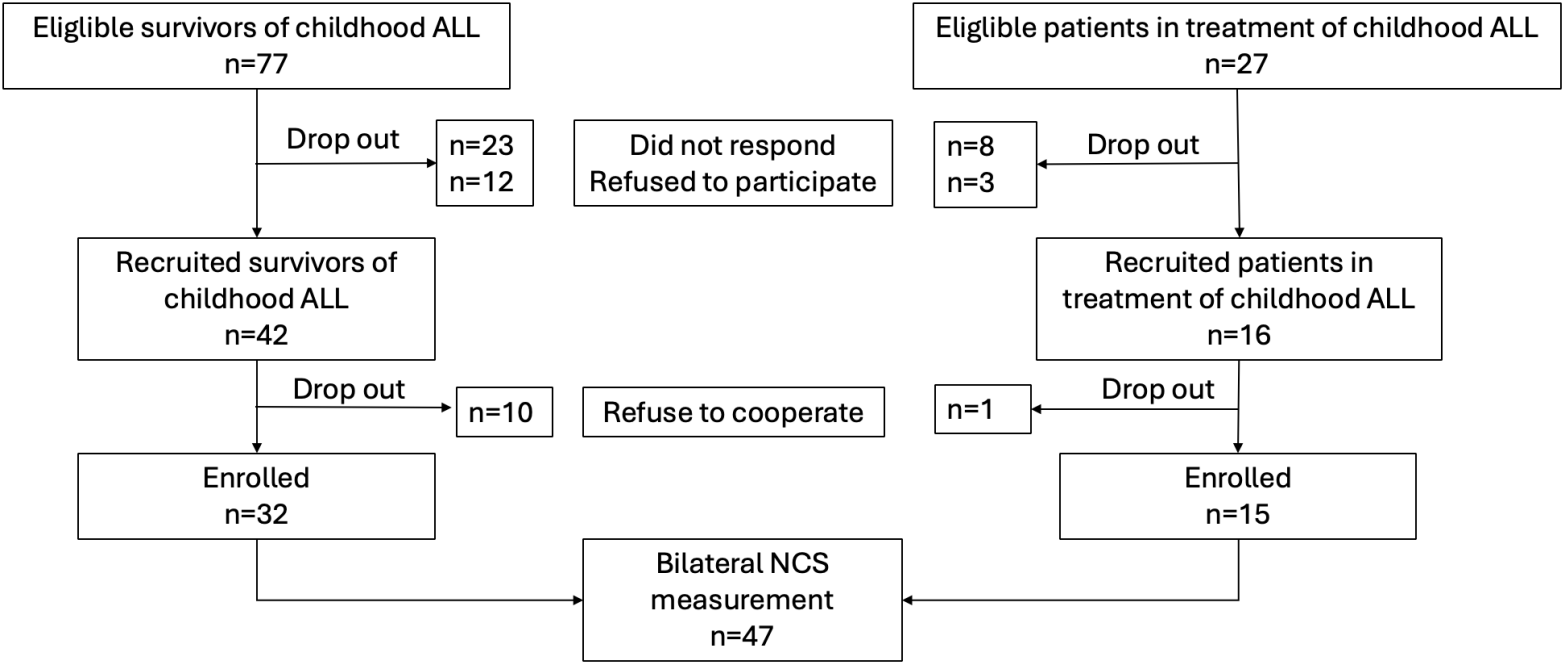
Flowchart of patient recruitment.

Of 77 eligible post-treatment survivors, 32 were successfully recruited. Of 27 eligible patients undergoing active chemotherapy, 15 were enrolled. Following the methodological decision to include only one independent observation per participant, the final analyzed dataset comprised a total of 47 independent bilateral NCS assessments (32 from post-treatment survivors and 15 from patients in active treatment). See Fig 1 for the participant flow diagram.

The study followed the Declaration of Helsinki and was approved by the Ethics Committee of the Third Faculty of Medicine, Charles University, Prague (approval date 11 November 2020, number 388821). Written informed consent was obtained from participants aged 18 and older or from parents or legal guardians of minors prior to assessment.

### 2.2 Procedures and measurements

Participants were assessed during routine ALL follow-up visits. Post-treatment survivors underwent a single, one-time nerve conduction study (NCS). Patients on active chemotherapy were originally scheduled for two assessments to align with routine oncology visits: the first during Week 8 (days 56–64 of Protocol I) and the second during Week 21 (days 49–56 of Protocol M). However, as detailed above, only data from the Week 21 assessment—representing identical cumulative vincristine exposure but allowing sufficient time for the neurotoxic phenotype to fully manifest—were included in the current analysis to maintain data independence.

Clinical data were extracted from medical records prior to testing, including age at diagnosis and assessment, height and weight at first assessment, treatment protocol, and cumulative vincristine dose. All participants received the same antifungal and antibiotic prophylaxis, compatible with vincristine [32].

Bilateral NCS were performed to assess CMAP amplitude, latency, and conduction velocity in peroneal (extensor digitorum brevis), tibial (abductor hallucis), median (abductor pollicis brevis), and ulnar (abductor digiti minimi) nerves. The examiner was blinded to clinical status, and room temperature was maintained between 20 and 25 °C.

SNAP parameters were measured antidromically in the sural nerves bilaterally, with electrodes on the lateral ankle and stimulation 14 cm proximally [33]. Ground electrodes were placed between stimulation and recording sites, with impedance below 5 kiloohms. Orthodromic SNAPs were recorded in median, ulnar, and superficial radial nerves bilaterally, using ring electrodes for finger stimulation and wrist recordings. Superficial radial stimulation was applied ~10 cm distal to the forearm recording site [34]. All recordings used surface electrodes; limb temperatures were maintained between 32 and 36 °C.

For motor nerves, amplitude (in millivolts) was recorded from proximal stimulation, distal latency was the time to CMAP onset (ms), and conduction velocity was calculated from stimulation site distances divided by latency differences (m/s). Stimulation was applied at the wrist and elbow (upper limbs) or ankle and knee (lower limbs).

For sensory nerves, amplitude was peak-to-peak (μV), distal latency was peak latency (ms), and conduction velocity was calculated as for motor nerves.

The ped-mTNS was used to clinically assess sensory, motor, and autonomic symptoms. A score of 5 or more (out of 32) indicated VIPN [35,36]. In this study, the ped-mTNS estimated clinical VIPN prevalence but was not used in statistical analyses or compared with NCS data.

### 2.3 Normative reference values and definition of abnormality

Age-adjusted normative values were taken from a large pediatric cohort study providing age-stratified means and SDs for motor and sensory nerves from 198 healthy children (birth to 18 years) across ten age strata [33]. Each participant was matched to the corresponding age group. Following the electrodiagnostic criteria for distal symmetric polyneuropathy [29], abnormalities were defined as values outside the 1st–99th percentile, operationalized as mean ± 2.3 SD from the age-specific reference data [33]. This normative dataset was selected due to its methodological consistency with our protocol, including identical temperature requirements and standardized amplitude measurement techniques (baseline-to-peak for CMAP and peak-to-peak for SNAP) [33].

For median and ulnar SNAPs and superficial radial nerve, published normative values were not directly applicable due to different stimulation techniques. In the reference study [33], median and ulnar SNAPs were reported using antidromic digit-to-wrist recordings, whereas our study used orthodromic digit-to-wrist recordings. For the superficial radial nerve, only antidromic values were available, while we employed an orthodromic forearm-to-wrist technique. Because these approaches yield systematically different amplitudes, these nerves were not dichotomized and were analyzed as continuous variables (ICC) only.

For limb-level DSP classification, we applied the England et al. (2005) criterion requiring abnormalities in at least two distinct nerves on the same side, including sural involvement.

### 2.4 Statistical analyses

The primary outcome was the degree of electrophysiological asymmetry between the left and right sides, evaluated at three hierarchical levels.

First, continuous nerve conduction parameters were compared between sides for each nerve assessed using the intraclass correlation coefficient (ICC) from random effects models [37–39]. This approach accounts for the paired data structure and quantifies the variance due to individual differences rather than measurement error. ICC values range from 0 to 1, with higher values indicating stronger inter-side agreement [40]. Ninety-five percent confidence intervals were also reported to evaluate the precision and clinical relevance of ICC estimates [39]. Interpretation thresholds were <0.50 poor, 0.50 to 0.75 moderate, 0.75 to 0.90 good, and >0.90 excellent reliability.

Second, Cohen’s kappa was calculated for parameters with available normative thresholds to assess binary agreement with agreement interpretation as <0.00 poor, 0.00 to 0.20 slight, 0.21 to 0.40 fair, 0.41 to 0.60 moderate, 0.61 to 0.80 substantial, and 0.81 to 1.00 almost perfect [41]. McNemar’s test was applied to measure statistical significance [42] with ten or more discordant observations present. A p-value below 0.05 indicated significant lateral asymmetry.

At the nerve level, each nerve was labeled normal or abnormal if at least one parameter (latency, amplitude, or velocity) was outside the normative range. Cohen’s kappa and McNemar’s test were again used.

Finally, same approach was used to assess side agreement in neuropathy.

## 3 Results

Sample characteristics are shown in Table 1. The cohort included 47 bilateral NCS (32 in post-treatment survivors and 15 in patients on active therapy). The mean age at diagnosis was 7.4 years (SD 4.2), and the mean age at the time of nerve conduction assessment was 9.7 years (SD 3.9). In the overall sample, boys were more represented than girls, with a total male-to-female ratio of 26:21. The cohort included predominantly children with B-cell precursor ALL (n = 43), while the T-cell phenotype was less frequent (n = 4). Clinical signs of neuropathy, defined by a ped-mTNS score ≥ 5, were present in 14 participants (29.8%), with slightly higher scores observed in patients on treatment. Cumulative vincristine dose differed markedly between groups, averaging 13.4 mg/m² in survivors and 6.4 mg/m² in the treatment subgroup, reflecting differences in protocol exposure and timing of assessments.

**Table 1:** Demographic, treatment and clinical characteristics of the study cohort.

Variable	Overall, n = 47	Children off therapy, n = 32	Children on therapy, n = 15
**Age at dg. (years)**	7.4 ± 4.2 (1.3–16.9)	6.3 ± 3.8 (1.3–16.6)	9.7 ± 4.3 (4.8–16.9)
**Age at assessment (years)**	9.7 ± 3.9 (4.5–18.8)	9.6 ± 3.8 (4.5–18.8)	10.0 ± 4.3 (5.2–17.2)
**Weight at time of assessment (kg)**	36.9 ± 17.1 (15.2–72.0)	36.2 ± 16.3 (15.2–68.0)	38.5 ± 19.2 (18.0–72.0)
**Height at time of assessment (cm)**	137.6 ± 23.2 (95.0–185.0)	135.6 ± 19.8 (95.0–163.5)	141.6 ± 29.5 (106.0–185.0)
**BMI at time of assessment**	18.4 ± 3.8 (13.4–28.3)	18.6 ± 4.0 (13.4–28.3)	18.0 ± 3.4 (14.5–24.6)
**BMI percentile**	60.6 ± 30.4 (0.9–99.9)	63.1 ± 29.5 (3.8–99.9)	55.3 ± 32.8 (0.9–98.0)
**Years from last VCR to date of assessment**	1.9 ± 1.4 (0.1–5.0)	2.6 ± 1.1 (1.1–5.0)	0.3 ± 0.1 (0.1–0.5)
**Years from end of maintenance therapy**	1.4 ± 1.1 (0.1–3.5)	1.4 ± 1.1 (0.1–3.5)	NA
**Cumulative dose VCR (mg/m**^**2**^)	11.2 ± 4.1 (6.0–18.0)	13.4 ± 2.9 (6.0–18.0)	6.4 ± 1.1 (6.0–9.0)
**Ped-mTNS score**	4.4 ± 4.3 (0.0–21.0)	4.2 ± 5.0 (0.0–21.0)	4.8 ± 2.5 (1.0–10.0)
**Girls/Boys**	21/26	16/16	5/10
**BCP-ALL/T-ALL**	43/4	31/1	12/3
**Neuropathy, n yes**	14	7	7
**High risk, n yes**	11	9	2
**CNS status 2, n yes**	5	4	1

Data are presented as mean ± SD (range) for continuous variables and n for categorical variables. BMI = body mass index; VCR = vincristine; BCP-ALL = B-cell precursor acute lymphoblastic leukemia; T-ALL = T-cell acute lymphoblastic leukemia; Ped-mTNS = Pediatric-modified Total Neuropathy Score; CNS = central nervous system. Neuropathy was defined as ped-mTNS ≥ 5.

A total of 47 bilateral NCS were included in the analysis. The distribution of abnormal findings across individual nerve conduction parameters and the proportion of whole-nerve abnormalities by side are summarized in Table 2.

**Table 2:** Summary of abnormal findings in nerve conduction parameters and whole-nerve abnormality rates by side.

Nerve	Amplitude	CV	Latency	whole nerve % abnormal right	whole nerve % abnormal left
Tibial	28	9	8	29.8%	40.4%
Peroneal	21	9	4	21.3%	34.0%
Median	22	7	6	27.7%	21.3%
Ulnar	20	14	0	21.3%	42.6%
Sural	0	2	3	2.1%	4.3%

The table displays the total number of abnormal findings for each nerve conduction parameter—amplitude, conduction velocity (CV), and distal latency—across five bilaterally tested nerves, including four motor nerves (tibial, peroneal, median, and ulnar) and one sensory nerve (sural). Values represent the total number of abnormal findings across both sides (right and left). Percentages under “whole nerve % abnormal right/left” represent the proportion of right and left sides, respectively, classified as abnormal based on at least one parameter exceeding predefined pathological thresholds. A total of 94 nerves (from 47 participants, both sides per nerve) were assessed for all included nerves.

Inter-side agreement was evaluated at three levels: (1) side-to-side comparison of individual NCS parameters, (2) inter-side agreement in whole-nerve classification, and (3) side-level diagnosis of NCS neuropathy. Complete results for all evaluated parameters, including inter-side agreement statistics, are presented in Table 3.

**Table 3:** Agreement between right and left nerve conduction parameters: classification-based (Cohen’s κ) and value-based (ICC) comparison, and inter-side asymmetry (McNemar test).

Nerve	N	Kappa	Strength of agreement	McNemar p-value	ICC [95% CI]	ICC p-value
**Tibial motor**	47	.49	moderate	.23	--	--
Latency	47	.73	substantial	X	.07 [−.21–.34]	.32
Amplitude	47	.40	fair	X	.55 [.31–.72]	<.001
CV	47	.39	fair	X	.62 [.41–.77]	<.001
**Peroneal motor**	47	.38	fair	.15	--	--
Latency	47	.48	moderate	X	.57 [.34–.73]	<.001
Amplitude	47	.09	slight	X	.35 [.08–.58]	.01
CV	47	.63	substantial	X	.64 [.43–.79]	<.001
**Median motor**	47	.48	moderate	.50	--	--
Latency	47	.64	substantial	X	.80 [.66–.88]	<.001
Amplitude	47	.53	moderate	X	.63 [.42–.78]	<.001
CV	47	.54	moderate	X	.40 [.12–.61]	<.001
**Ulnar motor**	47	.44	moderate	.01	--	--
Latency	47	--	--	X	.62 [.38–.78]	<.001
Amplitude	47	.50	moderate	X	.64 [.43–.79]	<.001
CV	47	.19	slight	X	.28 [.02–.52]	.02
**Median sensory**	--	--	--	--	--	--
Latency	45	--	--	--	.90 [.82–.94]	<.001
Amplitude	45	--	--	--	.30 [.03–.54]	.01
CV	--	--	--	--	--	--
**Ulnar sensory**	--	--	--	--	--	--
Latency	43	--	--	--	.92 [.87–.96]	<.001
Amplitude	43	--	--	--	.62 [.40–.78]	<.001
CV	--	--	--	--	--	--
**Radial sensory**	--	--	--	--	--	--
Latency	38	--	--	--	.93 [.88–.96]	<.001
Amplitude	38	--	--	--	.49 [.21–.70]	<.001
CV	36	--	--	--	.59 [.33–.77]	<.001
**Sural sensory**	47	.66	substantial	1.00	--	--
Latency	47	.66	substantial	X	.94 [.90–.97]	<.001
Amplitude	47	--	--	X	.59 [.36–.75]	<.001
CV	47	1.00	almost perfect	X	.68 [.49–.81]	<.001
**NCS-defined polyneuropathy**	47	.66	substantial	1.00	--	--

Strength of agreement was classified according to standard Cohen’s κ interpretation guidelines. McNemar p-value indicates statistical significance of side-to-side asymmetry in abnormality frequency. McNemar’s test was only interpreted for parameters with ≥10 discordant pairs. For parameters with insufficient discordant cases, McNemar values are not reported (X). ICC (intraclass correlation coefficient, model 2) represents the degree of inter-side agreement in continuous parameter values. ICC p-value indicates the statistical significance of the ICC estimate. CI = Confidence Interval. CV = conduction velocity. The number of observations (N) may vary between nerves due to missing or technically unobtainable measurements. “--” indicates that normative reference values required for dichotomous classification of abnormality were not available for this parameter, although bilateral measurements were performed and ICC was computed.

### 3.1 Side-to-side comparison of individual NCS parameters

The first analysis level assessed inter-side agreement of continuous nerve conduction parameters using ICC, Cohen’s kappa, and McNemar’s test. Most parameters showed only moderate agreement, suggesting limited bilateral symmetry in peripheral nerve function.

According to ICC, no motor parameter met the threshold for good reliability (ICC ≥ 0.75). Several showed poor agreement (ICC < 0.50), including tibial latency (ICC = 0.07, CI − .21 to .34, p = .32), peroneal amplitude (ICC = 0.35, CI .08 to .58, p = .01), median conduction velocity (ICC = 0.40, CI .12 to .61, p < .001), and ulnar conduction velocity (ICC = 0.28, CI .02 to .52, p = .02). In contrast, sensory distal latencies in the median, ulnar, radial, and sural nerves showed excellent agreement (ICC ≥ 0.90, all ps < .001), indicating high bilateral symmetry in sensory conduction timing.

Cohen’s kappa values supported these results at the categorical level. Motor parameters showed variable categorical agreement. Tibial latency (kappa = 0.73) and median latency (kappa = 0.64) demonstrated substantial agreement, while most other parameters, such as tibial amplitude (kappa = 0.40) and peroneal latency (kappa = 0.48), showed fair to moderate agreement. Peroneal amplitude (kappa = 0.09) and ulnar conduction velocity (kappa = 0.19) showed only slight agreement. In contrast, sural conduction velocity demonstrated perfect categorical agreement (kappa = 1.00). McNemar’s test results for individual parameters were limited by the low number of discordant pairs (fewer than 10 in all individual parameters), therefore specific lateral trends at the parameter level could not be statistically confirmed.

In summary, sensory conduction latencies were highly symmetric, while motor axonal parameters displayed notable asymmetry.

### 3.2 Inter-side agreement in whole-nerve classification

When each nerve was classified as abnormal if any parameter exceeded pathological thresholds, inter-side agreement was moderate for the tibial (kappa = 0.49), median (kappa = 0.48), and ulnar (kappa = 0.44) nerves, and fair for the peroneal nerve (kappa = 0.38). Sural whole-nerve agreement was substantial (kappa = 0.66). Notably, McNemar’s test revealed a statistically significant lateral asymmetry in abnormality frequency for the whole ulnar nerve (p = .01). As shown in Table 2, the left ulnar nerve was classified as abnormal exactly twice as often as the right ulnar nerve (42.6% vs. 21.3%).

### 3.3 Inter-side agreement in NCS polyneuropathy classification

When each limb was independently assessed for NCS-defined polyneuropathy using standard electrodiagnostic criteria, inter-side agreement was substantial (kappa = 0.66). However, sural nerve abnormalities were rare overall, which inherently limited the number of cases meeting the full DSP criteria, as sural involvement is a prerequisite. Consequently, due to the very low number of positive polyneuropathy cases, McNemar’s test (p = 1.00) did not detect any significant lateral asymmetry at the generalized polyneuropathy level.

## 4 Discussion

To our knowledge this study is among the first to systematically analyze electrophysiological asymmetry in pediatric ALL patients treated with vincristine. Using continuous and categorical nerve conduction data, we found consistent inter-side differences. Limb agreement was at best moderate, regardless of analysis method. These findings do not fully align with the assumption that VIPN is a symmetric neuropathy.

The mean ped-mTNS score in our cohort was 4.4 (SD 4.3), ranging from 0 to 21, indicating substantial clinical variability. Similar variability was reported in other studies: 2–19 (median 9) [36], 0–21 (mean 4.4) [43], and 2–18 (median 10) [44]. Using the threshold of 5 or more, clinical neuropathy was present in 29.8 percent of the cohort and 46.7 percent of those in treatment. These rates are lower than the 86.6 percent reported by Gilchrist et al. whose patients had higher vincristine exposure (15.9 ± 8.0 mg/m^2^). This supports the link between vincristine dose and neuropathy severity [7,11,45,46] and is consistent with the comparability of our sample.

Abnormalities were more frequent in motor nerves. This aligns with reports of motor-predominant neuropathy in children treated with vincristine [9,16–18,24–26]. CMAP amplitude was the most affected parameter, consistent with previous work identifying it as a hallmark of VIPN [4,15,19]. Although this suggests motor involvement, broader sensory testing might have revealed additional SNAP abnormalities beyond the sural nerve.

Sensory latencies showed high inter-side agreement, while CMAP amplitude and velocity showed only poor to moderate agreement. Whole nerve abnormality and polyneuropathy classifications also had limited agreement, highlighting the need for bilateral assessment. Previous studies rarely included formal asymmetry analyses. Jain et al. [25] reported 86.4% symmetric neuropathy without defining asymmetry. Viinikainen et al. [14] found bilaterally reduced peroneal and sural amplitudes supporting a predominantly symmetric pattern, but noted an upper-limb-dominant subgroup. Courtemanche et al. [27], using an amplitude ratio <50%, found asymmetry in 4 of 6 children (66.7%).

Data from other neuropathies provide context. Pediatric diabetic polyneuropathy is considered symmetric [47,48]. Abuelwafaa et al. [49] found similar side abnormality rates as our study but no asymmetry analysis. We observed comparable side differences but only fair to moderate agreement (kappa 0.38–0.49), exposing the limits of descriptive approaches. In adult DSP, bilateral symmetry is well established. High ICC and kappa values for sural (0.94/0.97), peroneal (0.75/0.68), and tibial amplitudes (0.85/0.74) were reported. Our values were markedly lower: ICCs of 0.59 (sural), 0.35 (peroneal), and 0.55 (tibial), with kappa values of 0.09 (peroneal) and 0.40 (tibial). This discrepancy reflects fundamental phenotypic differences: DSP is classically symmetric and length-dependent [29,50], with sensory symptoms predominating over motor involvement [51], whereas pediatric VIPN is typically motor-predominant [14,16–18] and may deviate from the classical length-dependent distribution, particularly in younger patients [23]. In healthy individuals, conduction parameters are highly symmetric [52–55]. Amplitude ratios below 0.2 are rare, and values under 0.5 are considered abnormal [55]. In our study, 40.4 percent had peroneal amplitude ratios below 0.5, and 10.6 percent below 0.2. For tibial nerves, 19.1 percent were below 0.5 and 2.1 percent below 0.2 (S1 Table). These rates exceed the expected physiologic range, supporting the notion that vincristine-induced neuropathy may produce asymmetrical axonal involvement that could be underestimated in unilateral or non-quantitative protocols.

Our NCS findings in vincristine-exposed children suggest that VIPN in this population may not be consistently symmetric and can present with side-to-side asymmetry. This observation is clinically relevant because postural and balance deficits are well documented in ALL survivors and have been linked to vincristine-related peripheral neuropathy disrupting somatosensory input [56]. Peripheral neuropathy has also been associated with impaired dynamic mobility and endurance, limiting functional capacity [57,58]. Systematic reviews highlight that balance impairments are frequent during and after therapy, may persist into adulthood, and can negatively affect motor development, mobility, and quality of life [5]. The persistence of these treatment-related neurotoxic effects as significant long-term outcomes is increasingly recognized; for example, recent data from adult cohorts demonstrate that VIPN symptoms can remain a chronic burden for years after therapy completion [59]. Neuromuscular mechanisms, including reduced tibialis anterior activation, poor balance scores, and lower jump performance, further support the impact of neuropathy on participation and quality of life in survivors [60]. Mechanistic studies also indicate that vincristine contributes to motor unit loss and delayed muscle regeneration, potentially reinforcing long-term motor dysfunction [61]. Our findings extend these observations by showing that vincristine-related neuropathy in children is often asymmetric at the electrophysiological level. Such asymmetry may underlie part of the postural imbalance, gait deviations, and musculoskeletal complaints described in survivors, and may warrant systematic assessment; future studies should test whether targeted rehabilitation improves outcomes

Case reports further illustrate the diagnostic relevance of asymmetry. Unilateral foot drop [23], limb weakness [26], and phrenic [62] or cranial nerve palsies [63–65] have been described in vincristine-treated children, which could be missed by unilateral testing. One report even described asymmetric peroneal findings that led to the diagnosis of Charcot–Marie–Tooth 1A, previously unrecognized and unmasked by vincristine [66]. Though rare, such examples underscore the potential diagnostic value of bilateral testing.

The use of adult DSP criteria in pediatric VIPN remains limited. DSP is defined by symmetric, length-dependent, sensory-predominant findings with sural involvement [29,67]. In our cohort, sural abnormalities were rare, even when other nerves were affected. Prior studies confirm low sural sensitivity in children [17,18,25,26,68]. Requiring sural abnormality may contribute to underrecognition of pediatric VIPN. A more suitable pediatric approach might include abnormalities in two or more nerves on one side, regardless of sural involvement. Including motor and sensory fibers from both limbs may improve diagnostic sensitivity. Though hypothetical, this model reflects our findings and could inform future criteria.

Several mechanisms may explain the observed asymmetry. Regional differences in vincristine uptake and axonal transport have been proposed and could contribute to uneven axonal injury [69,70], which may help explain the poor interside agreement in peroneal CMAP and the moderate values in tibial and median nerves. Vascular variability, including differences in nerve perfusion and blood-nerve barrier integrity [71], may increase vulnerability in less vascularized regions and contribute to asymmetric or mononeuritis multiplex like patterns [27]. Immune mediated inflammation, though not assessed here, may also damage nerves and disrupt the blood-nerve barrier [22,72], helping explain low agreement in motor parameters.

Our findings suggest that unilateral NCS may miss neuropathy in children with asymmetric involvement. Bilateral testing is advisable when asymmetry is suspected or for monitoring progression, while amplitude should be interpreted with caution in routine care.

Strengths include a well-characterized cohort, standardized bilateral NCS, and to our knowledge, among the first multi-level asymmetry analysis in this context. Several limitations of this study warrant consideration. First, we enrolled vincristine-exposed children regardless of clinical symptoms, which may have led to a more heterogeneous cohort than one composed exclusively of symptomatic patients. Second, the primarily cross-sectional design necessitated by our focus on statistical independence of observations for the primary analysis limits our ability to definitively track the evolution of asymmetry. Third, our choice of conservative abnormality thresholds (±2.3 SD) may have resulted in the underestimation of milder asymmetric cases. Additionally, the limited applicability of normative data for certain sensory nerves and the inherent inability of the ped-mTNS to capture side-to-side differences remain notable constraints in the comprehensive assessment of asymmetry. Future research should prioritize longitudinal designs to determine whether the active-treatment phenotype differs meaningfully from the survivor phenotype. Investigating how patterns of NCS abnormalities shift as vincristine dosages accumulate will be crucial for understanding the temporal development of electrophysiological asymmetry. Furthermore, future studies should combine bilateral NCS with side-specific functional and biomechanical measures to clarify the clinical relevance of these asymmetries for long-term postural control, gait, and overall motor outcomes in this pediatric population.

Overall, our results indicate that pediatric VIPN does not consistently follow a symmetric pattern. Electrophysiological asymmetry was frequent and may contribute to postural imbalance, gait deviations, and musculoskeletal complaints described in survivors. Systematic detection of asymmetry may be warranted, both to avoid under- or overestimation of neuropathy and to guide targeted rehabilitation aimed at preventing secondary postural problems and supporting long-term motor outcomes and quality of life.

## Supporting information

S1 Table Inter-side amplitude ratios across examined nerves. This table provides detailed calculations of side-to-side amplitude ratios for all assessed motor and sensory nerves, including percentages of nerves exceeding the 0.5 and 0.2 asymmetry thresholds.
(DOCX)

S2 Dataset The minimal data set file. (XLSX)
