# Supplementary material for: Electrophysiological asymmetry in vincristine-exposed children with acute lymphoblastic leukemia: Evidence from bilateral nerve conduction studies
Source: PLoS One. 2026 Jun 29;21(6):e0352440. doi: 10.1371/journal.pone.0352440 (PMC13313357; doi:10.1371/journal.pone.0352440)
Supplement: S1 Table — This table provides detailed calculations of side-to-side amplitude ratios for all assessed motor and sensory nerves, including percentages of nerves exceeding the 0.5 and 0.2 asymmetry thresholds. (DOCX) [file pone.0352440.s001.docx]

Supplementary Table S1. Inter-side amplitude ratios across examined nerves

| **Nerve** | **Total number of bilateral recordings** | **Number of cases with ratio < 0.5** | **Percentage with ratio < 0.5 (%)** | **Number of cases with ratio < 0.2** | **Percentage with ratio < 0.2 (%)** |
| --- | --- | --- | --- | --- | --- |
| Tibial motor | 47 | 9 | 19.1 | 1 | 2.1 |
| Peroneal motor | 47 | 19 | 40.4 | 5 | 10.6 |
| Median motor | 46 | 7 | 15.2 | 0 | 0.0 |
| Ulnar motor | 47 | 1 | 2.1 | 0 | 0.0 |
| Median sensory | 45 | 8 | 17.8 | 1 | 2.2 |
| Ulnar sensory | 43 | 6 | 14.0 | 0 | 0.0 |
| Radial sensory | 38 | 10 | 26.3 | 0 | 0.0 |
| Sural sensory | 47 | 3 | 6.4 | 1 | 2.1 |

Amplitude ratios were calculated as the ratio of the smaller to the larger amplitude between sides . Only valid bilateral recordings (> 0) were included . Thresholds of < 0.5 and < 0.2 were used to summarize marked inter-side asymmetry; based on established physiological limits, such pronounced differences are rare in healthy individuals and strongly suggest pathological nerve involvement (Bromberg and Jaros 1998)
