# Supplementary material for: Electrophysiological asymmetry in vincristine-exposed children with acute lymphoblastic leukemia: Evidence from bilateral nerve conduction studies
Source: PLoS One. 2026 Jun 29;21(6):e0352440. doi: 10.1371/journal.pone.0352440 (PMC13313357; doi:10.1371/journal.pone.0352440)
Supplement: Attachment — Submitted filename: Response to Reviewers.pdf [file pone.0352440.s003.pdf]

April 12, 2026

To: Ali M. KH. Al-Salahat, M.B.Ch.B.

Academic Editor

PLOS One

Dear Dr. Ali M. KH. Al-Salahat:

We would like to thank you and the reviewers for the thorough and constructive evaluation of our manuscript titled "Electrophysiological Asymmetry in Vincristine-Exposed Children with Acute Lymphoblastic Leukemia: Evidence from Bilateral Nerve Conduction Studies" (PONE-D-25-47490).

We greatly appreciate the insightful comments, particularly regarding the statistical methodology, which have significantly strengthened the quality and clinical relevance of our paper. We have carefully addressed all comments point-by-point below. Changes in the revised manuscript are tracked.

Journal Requirements & Editor Comments:

- **Formatting:** We have reviewed and updated the manuscript to ensure it fully meets PLOS ONE's style requirements.
- **Funding Information:** We have corrected the discrepancy between the 'Funding Information' and 'Financial Disclosure' sections. The grant numbers are now accurate and match perfectly.
- **Supporting Information:** We have included captions for our Supporting Information files at the end of the manuscript and updated all in-text citations accordingly.
- **Data Anonymization:** As requested by the editorial office, the uploaded minimal dataset (S2_Minimal_Dataset.xlsx) has been fully anonymized. Exact ages have been rounded to whole numbers and time-from-treatment intervals to a single decimal place to ensure participant privacy while preserving the scientific reproducibility of our age-stratified data.

Responses to Reviewer #1

Reviewer #1: *Nicely framed and meticulously performed study. No novel findings from the study.*

Response: We thank the reviewer for the positive appraisal of our study design and methodology. Regarding the novelty of our findings, we would like to respectfully highlight the primary contribution of our work.

The traditional clinical consensus, which is also reflected in the excellent review by Li et al. (2020) that you recommended, characterizes VIPN as a strictly symmetric neuropathy. The primary novelty of our study lies in systematically challenging this long-standing assumption. To our knowledge, this is the first study to provide quantitative evidence of significant electrophysiological asymmetry across multiple nerves in a pediatric ALL cohort. We believe that demonstrating this distinct, asymmetric pattern introduces a paradigm shift in how VIPN should be assessed, adding a highly novel and clinically relevant perspective to the existing literature.

Reviewer #1: *I would like to refer to following articles as well: Li, T., et al. (2024)... and Guizhou Li, et al. (2020)...*

Response: We appreciate the reviewer's suggestion. In fact, Li et al. (2020) was already a key reference in our original manuscript (Ref #20), as it represents the current clinical consensus. However, in the revised version, we have emphasized this reference even more in the Introduction (Lines [81 and 83]) to highlight that while such major reviews characterize VIPN as a strictly symmetric neuropathy, our study provides systematic evidence to the contrary. We believe this direct comparison clarifies the novelty of our findings. Additionally, we have now incorporated Li et al. (2024) (Ref #59), into the Discussion (Line [368]) to address long-term outcomes in survivors, further strengthening the manuscript.

Reviewer #1: *Also provide reference details used to compare for pediatric NCS testing.*

Response: We thank the reviewer for the opportunity to clarify the methodological basis of our comparative analysis. We have updated the section *"Normative reference values and definition of abnormality"* to include specific details regarding the reference study. We now explicitly state that our findings were compared against the normative data of Ryan et al. (2019), which involved a robust sample of 198 healthy children (99 males, 99 females) categorized into ten distinct age strata from birth to 18 years (Lines [181-183]). Furthermore, we have highlighted the methodological alignment between our study and the reference cohort regarding limb temperature maintenance and specific amplitude measurement techniques (baseline-to-peak for motor and peak-to-peak for sensory nerves), ensuring the validity of our findings (Lines [186-189]).

Responses to Reviewer #2

Reviewer #2: *The authors have chosen to include the repeated measures of the on-going treatment subgroup as independent observations, effectively doubling the contribution of this sub-group of patients to some of their statistical tests. This is an unsound choice. The statistical tests rely on the same assumptions of independence as other statistical methods, and clearly, the 26 of the observations coming from 13 patients makes these particular observations break that assumption. I understand that data about these patient cohorts is not easy to come by. However, the authors should choose ONE of the measurements, the same one in each patients (either the first, or the second) and stick with that one for their analyses. I might suggest the second measurement in each patient, since that is presumably the closest to the "survivor phenotype", but arguments could be made either way. Thus, they should change the data they present in tables 2 and 3 and reduce the N from 59 to 46. It is also somewhat misleading that in table 1, the N for the "children in therapy" group is presented as 27. I have read the text and I do understand that that number comes from 2 x 13 + 1, but nevertheless, it can be construed by more vicious reviewers as a dishonest way to present data. If they so wish, the authors could also keep their present results as supplementary material, but then they should also add a very clear defense of their choice of why they did so in the discussions. To my mind, this is the one big flaw that they need to address. At the very least, it should be clearly pointed out in the limitations paragraph.*

I would like to offer words of encouragement to the authors and once again say that their premise and overall results are interesting and warrant publishing. BUT this issue with the statistics must be solved before that.

Response: We sincerely thank the reviewer for identifying this critical methodological flaw. We fully agree that treating repeated measures as independent observations violated the assumptions of independence required for our statistical tests.

Following your recommendation, we have completely revised our analysis to include only one independent observation per patient. Regarding the choice of measurement for the treatment subgroup, we have selected the second assessment (Week 21) for all patients. While the cumulative vincristine dose is identical at both time points (as the final dose in this protocol phase is administered at Week 4), the second measurement at Week 21 allows sufficient time for the vincristine-induced peripheral neuropathy (VIPN) to fully manifest. By choosing this later time point, we provide a more accurate representation of the established neurotoxic phenotype, which, as you correctly noted, is more closely aligned with the "survivor phenotype."

Please note a minor correction regarding the final sample size: our cohort consists of 32 post-treatment survivors and 15 patients on active therapy (not 14 as previously implied by the math), bringing our total sample of independent observations to N = 47 (rather than 46).

We have completely recalculated Tables 1, 2, and 3 using this statistically sound dataset. To ensure full transparency and address your concern regarding the presentation of our data, Table 1 has been revised to reflect the exact number of unique participants (N = 15 for the therapy group), eliminating any misleading representation. We have also explicitly stated this methodological adjustment in the *Materials and Methods* section (Lines [108-117]).

Reviewer #2: *Furthermore, once this issue is resolved, it will also provide fertile grounds for a more in-depth exploration of important hypothese in the discussions section: "is the active treatment phenotype meaningfully different to the survivor phenotype?" and "might there be a changing pattern of NCS abnormalities as vincristine dosages accumulate during treatment?"*

Response: We highly value this insightful suggestion, as it points toward critical questions in the clinical management and understanding of VIPN. We completely agree that exploring the differences between the active-treatment and survivor phenotypes, as well as the longitudinal impact of cumulative vincristine dosage, represents a significant area for clinical investigation.

In the revised manuscript, we have chosen to maintain our primary focus on the systematic quantification of electrophysiological asymmetry. Therefore, we did not include new comparative statistical analyses between these subgroups. However, prompted by your comment, we have expanded our Limitations and Future Directions section (Lines [407-423) to explicitly integrate these hypotheses. Specifically, we have highlighted the necessity for future longitudinal studies to determine whether the active-treatment phenotype differs meaningfully from the survivor phenotype, and to track how patterns of NCS abnormalities shift as vincristine dosages accumulate. We believe that acknowledging these specific research questions provides a clear roadmap for subsequent studies and adds valuable clinical context to our conclusion.

Reviewer #2: *One other minor issues in rows 214 and 233 there is an error message "Error! Reference source not found.. "*

Response: We apologize for this formatting glitch. The error messages have been removed and correctly linked to the respective tables.

We would like to thank the Editor, and the two Reviewers for their highly constructive feedback, which we believe has helped us greatly improve the quality of the manuscript. We also hope the Editor and Reviewers now find the manuscript suitable for publication in *PLOS One*.

Yours sincerely,

prof. Alena Kobesova, MD, PhD

Corresponding author
