# Supplementary material for: Electrophysiological asymmetry in vincristine-exposed children with acute lymphoblastic leukemia: Evidence from bilateral nerve conduction studies
Source: PLoS One. 2026 Jun 29;21(6):e0352440. doi: 10.1371/journal.pone.0352440 (PMC13313357; doi:10.1371/journal.pone.0352440)
Supplement: Peer review history

# Peer review: Decision Letter 0

**DOI:** 10.1371/journal.pone.0352440.r001

**Authors:** Ali M. KH. Al-Salahat

3 Mar 2026

PONE-D-25-47490

Electrophysiological Asymmetry in Vincristine-Exposed Children with Acute Lymphoblastic Leukemia: Evidence from Bilateral Nerve Conduction Studies

PLOS One

Dear Dr. Kobesova,

Thank you for submitting your manuscript to PLOS ONE. After careful consideration, we feel that it has merit but does not fully meet PLOS ONE’s publication criteria as it currently stands. Therefore, we invite you to submit a revised version of the manuscript that addresses the points raised during the review process.

Editor comments:

Please carefully address the reviewers comments and follow the PLOS one author guidelines for your revised manuscript.

Please submit your revised manuscript by Apr 17 2026 11:59PM. If you will need more time than this to complete your revisions, please reply to this message or contact the journal office at plosone@plos.org. When you're ready to submit your revision, log on to https://www.editorialmanager.com/pone/ and select the 'Submissions Needing Revision' folder to locate your manuscript file.

Please include the following items when submitting your revised manuscript:

- A letter that responds to each point raised by the academic editor and reviewer(s). You should upload this letter as a separate file labeled 'Response to Reviewers'.
- A marked-up copy of your manuscript that highlights changes made to the original version. You should upload this as a separate file labeled 'Revised Manuscript with Track Changes'.
- An unmarked version of your revised paper without tracked changes. You should upload this as a separate file labeled 'Manuscript'.

If you would like to make changes to your financial disclosure, please include your updated statement in your cover letter. Guidelines for resubmitting your figure files are available below the reviewer comments at the end of this letter.

If applicable, we recommend that you deposit your laboratory protocols in protocols.io to enhance the reproducibility of your results. Protocols.io assigns your protocol its own identifier (DOI) so that it can be cited independently in the future. For instructions see: https://journals.plos.org/plosone/s/submission-guidelines#loc-laboratory-protocols. Additionally, PLOS ONE offers an option for publishing peer-reviewed Lab Protocol articles, which describe protocols hosted on protocols.io. Read more information on sharing protocols at https://plos.org/protocols?utm_medium=editorial-email&utm_source=authorletters&utm_campaign=protocols.

We look forward to receiving your revised manuscript.

Kind regards,

Ali M. KH. Al-Salahat, M.B.Ch.B.

Academic Editor

PLOS One

Journal Requirements:

When submitting your revision, we need you to address these additional requirements.

1. Please ensure that your manuscript meets PLOS ONE's style requirements, including those for file naming. The PLOS ONE style templates can be found at

https://journals.plos.org/plosone/s/file?id=wjVg/PLOSOne_formatting_sample_main_body.pdf and

https://journals.plos.org/plosone/s/file?id=ba62/PLOSOne_formatting_sample_title_authors_affiliations.pdf

2. We note that the grant information you provided in the ‘Funding Information’ and ‘Financial Disclosure’ sections do not match.

When you resubmit, please ensure that you provide the correct grant numbers for the awards you received for your study in the ‘Funding Information’ section.

3. Please include captions for your Supporting Information files at the end of your manuscript, and update any in-text citations to match accordingly. Please see our Supporting Information guidelines for more information: http://journals.plos.org/plosone/s/supporting-information.

4. If the reviewer comments include a recommendation to cite specific previously published works, please review and evaluate these publications to determine whether they are relevant and should be cited. There is no requirement to cite these works unless the editor has indicated otherwise.

Additional Editor Comments:

Reviewer #1:

Nicely framed and meticulously performed study.

No novel findings from the study.

I would like to refer to following articles as well

Li, T., Trinh, T., Bosco, A. et al. Characterising vincristine-induced peripheral neuropathy in adults: symptom development and long-term persistent outcomes. Support Care Cancer 32, 278 (2024). https://doi.org/10.1007/s00520-024-08484-5

Gui-zhou Li, Ya-hui Hu, De-yi Li, Yong Zhang, Hong-li Guo, Yun-man Li, Feng Chen, Jing Xu,

Vincristine-induced peripheral neuropathy: A mini-review,

NeuroToxicology,

Volume 81,

2020,

Pages 161-171,

ISSN 0161-813X,

https://doi.org/10.1016/j.neuro.2020.10.004.

Also provide reference details used to compare for pediatric NCS testing.

Reviewer #2:

This is an interesting manuscript concerning the NCS asymmetries in children wil ALL who have received vincristine. It is a valuable area of research since there is relatively little information to guide clinical management or patients' and families' expectations of VINP.

The manuscript is nicely written and the data concerning these patients is not easily obtained (due to the relative rarity of the disease and, especially in the author's geographic region, the relative paucity of complex interdisciplinary assessment opportunities compared to western institutions). Therefore, their work should be published, with one very big caveat.

The authors have chosen to include the repeated measures of the on-going treatment subgroup as independent observations, effectively doubling the contribution of this sub-group of patients to some of their statistical tests. This is an unsound choice. The statistical tests rely on the same assumptions of independence as other statistical methods, and clearly, the 26 of the observations coming from 13 patients makes these particular observations break that assumption.

I understand that data about these patient cohorts is not easy to come by. However, the authors should choose ONE of the measurements, the same one in each patients (either the first, or the second) and stick with that one for their analyses. I might suggest the second measurement in each patient, since that is presumably the closest to the "survivor phenotype", but arguments could be made either way. Thus, they should change the data they present in tables 2 and 3 and reduce the N from 59 to 46. It is also somewhat misleading that in table 1, the N for the "children in therapy" group is presented as 27. I have read the text and I do understand that that number comes from 2 x 13 + 1, but nevertheless, it can be construed by more vicious reviewers as a dishonest way to present data. If they so wish, the authors could also keep their present results as supplementary material, but then they should also add a very clear defense of their choice of why they did so in the discussions. To my mind, this is the one big flaw that they need to address. At the very least, it should be clearly pointed out in the limitations paragraph.

I would like to offer words of encouragement to the authors and once again say that their premise and overall results are interesting and warrant publishing. BUT this issue with the statistics must be solved before that.

Furthermore, once this issue is resolved, it will also provide fertile grounds for a more in-depth exploration of important hypothese in the discussions section: "is the active treatment phenotype meaningfully different to the survivor phenotype?" and "might there be a changing pattern of NCS abnormalities as vincristine dosages accumulate during treatment?". these patients are quite different from long term survivors and more interesting hypothese might come from these observations, if the data is treated correctly.

One other minor issues in rows 214 and 233 there is an error message "Error! Reference source not found.. " - I am assuming these should link to tables.

[Note: HTML markup is below. Please do not edit.]

Reviewers' comments:

Reviewer's Responses to Questions

**Comments to the Author**

1. Is the manuscript technically sound, and do the data support the conclusions?

The manuscript must describe a technically sound piece of scientific research with data that supports the conclusions. Experiments must have been conducted rigorously, with appropriate controls, replication, and sample sizes. The conclusions must be drawn appropriately based on the data presented.

Reviewer #1: Yes

Reviewer #2: Partly

**********

2. Has the statistical analysis been performed appropriately and rigorously?

Reviewer #1: Yes

Reviewer #2: No

**********

3. Have the authors made all data underlying the findings in their manuscript fully available?

The PLOS Data policy requires authors to make all data underlying the findings described in their manuscript fully available without restriction, with rare exception (please refer to the Data Availability Statement in the manuscript PDF file). The data should be provided as part of the manuscript or its supporting information, or deposited to a public repository. For example, in addition to summary statistics, the data points behind means, medians and variance measures should be available. If there are restrictions on publicly sharing data—e.g. participant privacy or use of data from a third party—those must be specified.

Reviewer #1: Yes

Reviewer #2: Yes

**********

4. Is the manuscript presented in an intelligible fashion and written in standard English?

PLOS ONE does not copyedit accepted manuscripts, so the language in submitted articles must be clear, correct, and unambiguous. Any typographical or grammatical errors should be corrected at revision, so please note any specific errors here.

Reviewer #1: Yes

Reviewer #2: Yes

**********

5. Review Comments to the Author

Please use the space provided to explain your answers to the questions above. You may also include additional comments for the author, including concerns about dual publication, research ethics, or publication ethics. (Please upload your review as an attachment if it exceeds 20,000 characters)

Reviewer #1: Nicely framed and meticulously performed study.

No novel findings from the study.

I would like to refer to following articles as well

Li, T., Trinh, T., Bosco, A. et al. Characterising vincristine-induced peripheral neuropathy in adults: symptom development and long-term persistent outcomes. Support Care Cancer 32, 278 (2024). https://doi.org/10.1007/s00520-024-08484-5

Gui-zhou Li, Ya-hui Hu, De-yi Li, Yong Zhang, Hong-li Guo, Yun-man Li, Feng Chen, Jing Xu,

Vincristine-induced peripheral neuropathy: A mini-review,

NeuroToxicology,

Volume 81,

2020,

Pages 161-171,

ISSN 0161-813X,

https://doi.org/10.1016/j.neuro.2020.10.004.

Also provide reference details used to compare for pediatric NCS testing.

Reviewer #2: This is an interesting manuscript concerning the NCS asymmetries in children wil ALL who have received vincristine. It is a valuable area of research since there is relatively little information to guide clinical management or patients' and families' expectations of VINP.

The manuscript is nicely written and the data concerning these patients is not easily obtained (due to the relative rarity of the disease and, especially in the author's geographic region, the relative paucity of complex interdisciplinary assessment opportunities compared to western institutions). Therefore, their work should be published, with one very big caveat.

The authors have chosen to include the repeated measures of the on-going treatment subgroup as independent observations, effectively doubling the contribution of this sub-group of patients to some of their statistical tests. This is an unsound choice. The statistical tests rely on the same assumptions of independence as other statistical methods, and clearly, the 26 of the observations coming from 13 patients makes these particular observations break that assumption.

I understand that data about these patient cohorts is not easy to come by. However, the authors should choose ONE of the measurements, the same one in each patients (either the first, or the second) and stick with that one for their analyses. I might suggest the second measurement in each patient, since that is presumably the closest to the "survivor phenotype", but arguments could be made either way. Thus, they should change the data they present in tables 2 and 3 and reduce the N from 59 to 46. It is also somewhat misleading that in table 1, the N for the "children in therapy" group is presented as 27. I have read the text and I do understand that that number comes from 2 x 13 + 1, but nevertheless, it can be construed by more vicious reviewers as a dishonest way to present data. If they so wish, the authors could also keep their present results as supplementary material, but then they should also add a very clear defense of their choice of why they did so in the discussions. To my mind, this is the one big flaw that they need to address. At the very least, it should be clearly pointed out in the limitations paragraph.

I would like to offer words of encouragement to the authors and once again say that their premise and overall results are interesting and warrant publishing. BUT this issue with the statistics must be solved before that.

Furthermore, once this issue is resolved, it will also provide fertile grounds for a more in-depth exploration of important hypothese in the discussions section: "is the active treatment phenotype meaningfully different to the survivor phenotype?" and "might there be a changing pattern of NCS abnormalities as vincristine dosages accumulate during treatment?". these patients are quite different from long term survivors and more interesting hypothese might come from these observations, if the data is treated correctly.

One other minor issues in rows 214 and 233 there is an error message "Error! Reference source not found.. " - I am assuming these should link to tables.

**********

6. PLOS authors have the option to publish the peer review history of their article (what does this mean?). If published, this will include your full peer review and any attached files.

If you choose “no”, your identity will remain anonymous but your review may still be made public.

**Do you want your identity to be public for this peer review?**  For information about this choice, including consent withdrawal, please see our Privacy Policy.

Reviewer #1: No

Reviewer #2: No

**********

[NOTE: If reviewer comments were submitted as an attachment file, they will be attached to this email and accessible via the submission site. Please log into your account, locate the manuscript record, and check for the action link "View Attachments". If this link does not appear, there are no attachment files.]

To ensure your figures meet our technical requirements, please review our figure guidelines: https://journals.plos.org/plosone/s/figures

You may also use PLOS’s free figure tool, NAAS, to help you prepare publication quality figures: https://journals.plos.org/plosone/s/figures#loc-tools-for-figure-preparation.

NAAS will assess whether your figures meet our technical requirements by comparing each figure against our figure specifications.

# Author comment: Author response to Decision Letter 1

**DOI:** 10.1371/journal.pone.0352440.r002

12 Apr 2026

Journal Requirements & Editor Comments:

• Formatting: We have reviewed and updated the manuscript to ensure it fully meets PLOS ONE's style requirements.

• Funding Information: We have corrected the discrepancy between the ‘Funding Information’ and ‘Financial Disclosure’ sections. The grant numbers are now accurate and match perfectly.

• Supporting Information: We have included captions for our Supporting Information files at the end of the manuscript and updated all in-text citations accordingly.

• Data Anonymization: As requested by the editorial office, the uploaded minimal dataset (S2_Minimal_Dataset.xlsx) has been fully anonymized. Exact ages have been rounded to whole numbers and time-from-treatment intervals to a single decimal place to ensure participant privacy while preserving the scientific reproducibility of our age-stratified data.

Responses to Reviewer #1

Reviewer #1: Nicely framed and meticulously performed study. No novel findings from the study.

Response: We thank the reviewer for the positive appraisal of our study design and methodology. Regarding the novelty of our findings, we would like to respectfully highlight the primary contribution of our work.

The traditional clinical consensus, which is also reflected in the excellent review by Li et al. (2020) that you recommended, characterizes VIPN as a strictly symmetric neuropathy. The primary novelty of our study lies in systematically challenging this long-standing assumption. To our knowledge, this is the first study to provide quantitative evidence of significant electrophysiological asymmetry across multiple nerves in a pediatric ALL cohort. We believe that demonstrating this distinct, asymmetric pattern introduces a paradigm shift in how VIPN should be assessed, adding a highly novel and clinically relevant perspective to the existing literature.

Reviewer #1: I would like to refer to following articles as well: Li, T., et al. (2024)... and Gui-zhou Li, et al. (2020)...

Response: We appreciate the reviewer's suggestion. In fact, Li et al. (2020) was already a key reference in our original manuscript (Ref #20), as it represents the current clinical consensus. However, in the revised version, we have emphasized this reference even more in the Introduction (Lines [81 and 83]) to highlight that while such major reviews characterize VIPN as a strictly symmetric neuropathy, our study provides systematic evidence to the contrary. We believe this direct comparison clarifies the novelty of our findings. Additionally, we have now incorporated Li et al. (2024) (Ref #59), into the Discussion (Line [368]) to address long-term outcomes in survivors, further strengthening the manuscript.

Reviewer #1: Also provide reference details used to compare for pediatric NCS testing.

Response: We thank the reviewer for the opportunity to clarify the methodological basis of our comparative analysis. We have updated the section "Normative reference values and definition of abnormality" to include specific details regarding the reference study. We now explicitly state that our findings were compared against the normative data of Ryan et al. (2019), which involved a robust sample of 198 healthy children (99 males, 99 females) categorized into ten distinct age strata from birth to 18 years (Lines [181-183]). Furthermore, we have highlighted the methodological alignment between our study and the reference cohort regarding limb temperature maintenance and specific amplitude measurement techniques (baseline-to-peak for motor and peak-to-peak for sensory nerves), ensuring the validity of our findings (Lines [186-189]).

Responses to Reviewer #2

Reviewer #2: The authors have chosen to include the repeated measures of the on-going treatment subgroup as independent observations, effectively doubling the contribution of this sub-group of patients to some of their statistical tests. This is an unsound choice. The statistical tests rely on the same assumptions of independence as other statistical methods, and clearly, the 26 of the observations coming from 13 patients makes these particular observations break that assumption.

I understand that data about these patient cohorts is not easy to come by. However, the authors should choose ONE of the measurements, the same one in each patients (either the first, or the second) and stick with that one for their analyses. I might suggest the second measurement in each patient, since that is presumably the closest to the "survivor phenotype", but arguments could be made either way. Thus, they should change the data they present in tables 2 and 3 and reduce the N from 59 to 46. It is also somewhat misleading that in table 1, the N for the "children in therapy" group is presented as 27. I have read the text and I do understand that that number comes from 2 x 13 + 1, but nevertheless, it can be construed by more vicious reviewers as a dishonest way to present data. If they so wish, the authors could also keep their present results as supplementary material, but then they should also add a very clear defense of their choice of why they did so in the discussions. To my mind, this is the one big flaw that they need to address. At the very least, it should be clearly pointed out in the limitations paragraph.

I would like to offer words of encouragement to the authors and once again say that their premise and overall results are interesting and warrant publishing. BUT this issue with the statistics must be solved before that.

Response: We sincerely thank the reviewer for identifying this critical methodological flaw. We fully agree that treating repeated measures as independent observations violated the assumptions of independence required for our statistical tests.

Following your recommendation, we have completely revised our analysis to include only one independent observation per patient. Regarding the choice of measurement for the treatment subgroup, we have selected the second assessment (Week 21) for all patients. While the cumulative vincristine dose is identical at both time points (as the final dose in this protocol phase is administered at Week 4), the second measurement at Week 21 allows sufficient time for the vincristine-induced peripheral neuropathy (VIPN) to fully manifest. By choosing this later time point, we provide a more accurate representation of the established neurotoxic phenotype, which, as you correctly noted, is more closely aligned with the "survivor phenotype."

Please note a minor correction regarding the final sample size: our cohort consists of 32 post-treatment survivors and 15 patients on active therapy (not 14 as previously implied by the math), bringing our total sample of independent observations to N = 47 (rather than 46).

We have completely recalculated Tables 1, 2, and 3 using this statistically sound dataset. To ensure full transparency and address your concern regarding the presentation of our data, Table 1 has been revised to reflect the exact number of unique participants (N = 15 for the therapy group), eliminating any misleading representation. We have also explicitly stated this methodological adjustment in the Materials and Methods section (Lines [108-117]).

Reviewer #2: Furthermore, once this issue is resolved, it will also provide fertile grounds for a more in-depth exploration of important hypothese in the discussions section: "is the active treatment phenotype meaningfully different to the survivor phenotype?" and "might there be a changing pattern of NCS abnormalities as vincristine dosages accumulate during treatment?"

Response: We highly value this insightful suggestion, as it points toward critical questions in the clinical management and understanding of VIPN. We completely agree that exploring the differences between the active-treatment and survivor phenotypes, as well as the longitudinal impact of cumulative vincristine dosage, represents a significant area for clinical investigation.

In the revised manuscript, we have chosen to maintain our primary focus on the systematic quantification of electrophysiological asymmetry. Therefore, we did not include new comparative statistical analyses between these subgroups. However, prompted by your comment, we have expanded our Limitations and Future Directions section (Lines [407-423) to explicitly integrate these hypotheses. Specifically, we have highlighted the necessity for future longitudinal studies to determine whether the active-treatment phenotype differs meaningfully from the survivor phenotype, and to track how patterns of NCS abnormalities shift as vincristine dosages accumulate. We believe that acknowledging these specific research questions provides a clear roadmap for subsequent studies and adds valuable clinical context to our conclusion.

Reviewer #2: One other minor issues in rows 214 and 233 there is an error message "Error! Reference source not found.. "

Response: We apologize for this formatting glitch. The error messages have been removed and correctly linked to the respective tables.

Attachment Submitted filename: Response to Reviewers.pdf

# Peer review: Decision Letter 1

**DOI:** 10.1371/journal.pone.0352440.r003

**Authors:** Ali M. KH. Al-Salahat

10 Jun 2026

Electrophysiological Asymmetry in Vincristine-Exposed Children with Acute Lymphoblastic Leukemia: Evidence from Bilateral Nerve Conduction Studies

PONE-D-25-47490R1

Dear Dr. Kobesova,

We’re pleased to inform you that your manuscript has been judged scientifically suitable for publication and will be formally accepted for publication once it meets all outstanding technical requirements.

Within one week, you’ll receive an e-mail detailing the required amendments. When these have been addressed, you’ll receive a formal acceptance letter and your manuscript will be scheduled for publication.

An invoice will be generated when your article is formally accepted. Please note, if your institution has a publishing partnership with PLOS and your article meets the relevant criteria, all or part of your publication costs will be covered. Please make sure your user information is up-to-date by logging into Editorial Manager at Editorial Manager® and clicking the ‘Update My Information' link at the top of the page. For questions related to billing, please contact billing support.

If your institution or institutions have a press office, please notify them about your upcoming paper to help maximize its impact. If they’ll be preparing press materials, please inform our press team as soon as possible -- no later than 48 hours after receiving the formal acceptance. Your manuscript will remain under strict press embargo until 2 pm Eastern Time on the date of publication. For more information, please contact onepress@plos.org.

Kind regards,

Tomoyoshi Komiyama, Ph.D

Academic Editor

PLOS One

Additional Editor Comments (optional):

Dear authors,

Thank you for submitting your revised manuscript.

I think it was much easier to understand than the original manuscript.

I am satisfied with the responses and the edits, so I am happy to accept this manuscript.

This manuscript will satisfy the readers' interest and provide valuable information to clinicians and researchers in this field.

The authors have addressed the reviewers’ comments appropriately, and the revisions have significantly improved the clarity and overall quality of the manuscript.

The study presents meaningful findings that contribute to our understanding of the topic and will be of interest to a broad readership.

The manuscript is generally well written.

As a minor point, I would encourage the authors to perform a final check for spelling, typographical errors, and consistency of terminology throughout the manuscript.

For instance, ped-mTNS is used in most sections, whereas Ped-mTNS appears on line 319.

Addressing these small inconsistencies would further enhance the quality of the paper.

Tomoyoshi Komiyama

Reviewers' comments:

Reviewer's Responses to Questions

**Comments to the Author**

1. If the authors have adequately addressed your comments raised in a previous round of review and you feel that this manuscript is now acceptable for publication, you may indicate that here to bypass the “Comments to the Author” section, enter your conflict of interest statement in the “Confidential to Editor” section, and submit your "Accept" recommendation.

Reviewer #2: All comments have been addressed

Reviewer #3: All comments have been addressed

**********

2. Is the manuscript technically sound, and do the data support the conclusions?

The manuscript must describe a technically sound piece of scientific research with data that supports the conclusions. Experiments must have been conducted rigorously, with appropriate controls, replication, and sample sizes. The conclusions must be drawn appropriately based on the data presented.

Reviewer #2: Yes

Reviewer #3: Yes

**********

3. Has the statistical analysis been performed appropriately and rigorously?

Reviewer #2: Yes

Reviewer #3: Yes

**********

4. Have the authors made all data underlying the findings in their manuscript fully available?

The PLOS Data policy requires authors to make all data underlying the findings described in their manuscript fully available without restriction, with rare exception (please refer to the Data Availability Statement in the manuscript PDF file). The data should be provided as part of the manuscript or its supporting information, or deposited to a public repository. For example, in addition to summary statistics, the data points behind means, medians and variance measures should be available. If there are restrictions on publicly sharing data—e.g. participant privacy or use of data from a third party—those must be specified.

Reviewer #2: Yes

Reviewer #3: Yes

**********

5. Is the manuscript presented in an intelligible fashion and written in standard English?

PLOS ONE does not copyedit accepted manuscripts, so the language in submitted articles must be clear, correct, and unambiguous. Any typographical or grammatical errors should be corrected at revision, so please note any specific errors here.

Reviewer #2: Yes

Reviewer #3: Yes

**********

6. Review Comments to the Author

Please use the space provided to explain your answers to the questions above. You may also include additional comments for the author, including concerns about dual publication, research ethics, or publication ethics. (Please upload your review as an attachment if it exceeds 20,000 characters)

Reviewer #2: Thank you for making the necessary changes to your statistical approach.

My concerns have been adequately addressed.

Please make sure to check for spelling/typing consistency, as there some minor issues. For example, ped-mTNS is variably referred to across the text as ped-mTNS (most places) and Ped-mTNS in others (line 319).

Other

Reviewer #3: This report by Jevic et al., titled “Electrophysiological Asymmetry in Vincristine-Exposed Children with Acute Lymphoblastic Leukemia: Evidence from Bilateral Nerve Conduction Studies,” presents the results of their retrospective investigation, which systematically analyzes electrophysiological asymmetry in a total of 47 pediatric ALL patients treated with vincristine. This is a well-organized study that succeeds in shedding light on the characteristics of vincristine‑induced peripheral neuropathy (VIPN) through systematic nerve conduction studies.

In actual clinical practice, there is often an implicit assumption that VIPN presents symmetrically, and as a result, clinicians may spend considerable time ruling out other conditions when encountering asymmetric or unilateral neurological symptoms. This study clearly demonstrates that VIPN can indeed manifest asymmetrically, providing valuable guidance for clinical decision‑making.

Since the authors have fully addressed all the concerns raised in the initial review, the revised manuscript is now suitable for publication in PLOS ONE.

**********

7. PLOS authors have the option to publish the peer review history of their article (what does this mean?). If published, this will include your full peer review and any attached files.

If you choose “no”, your identity will remain anonymous but your review may still be made public.

**Do you want your identity to be public for this peer review?**  For information about this choice, including consent withdrawal, please see our Privacy Policy.

Reviewer #2: No

Reviewer #3: **Yes:**  Atsushi Makimoto

**********

# Editor report: Acceptance letter

**DOI:** 10.1371/journal.pone.0352440.r004

**Authors:** Tomoyoshi Komiyama

PONE-D-25-47490R1

PLOS One

Dear Dr. Kobesova,

I'm pleased to inform you that your manuscript has been deemed suitable for publication in PLOS One. Congratulations! Your manuscript is now being handed over to our production team.

At this stage, our production department will prepare your paper for publication. This includes ensuring the following:

* All references, tables, and figures are properly cited

* All relevant supporting information is included in the manuscript submission,

* There are no issues that prevent the paper from being properly typeset

You will receive further instructions from the production team, including instructions on how to review your proof when it is ready. Please keep in mind that we are working through a large volume of accepted articles, so please give us a few days to review your paper and let you know the next and final steps.

Lastly, if your institution or institutions have a press office, please let them know about your upcoming paper now to help maximize its impact. If they'll be preparing press materials, please inform our press team within the next 48 hours. Your manuscript will remain under strict press embargo until 2 pm Eastern Time on the date of publication. For more information, please contact onepress@plos.org.

You will receive an invoice from PLOS for your publication fee after your manuscript has reached the completed accept phase. If you receive an email requesting payment before acceptance or for any other service, this may be a phishing scheme. Learn how to identify phishing emails and protect your accounts at https://explore.plos.org/phishing.

If we can help with anything else, please email us at customercare@plos.org.

Thank you for submitting your work to PLOS ONE and supporting open access.

Kind regards,

PLOS ONE Editorial Office Staff

on behalf of

Dr. Tomoyoshi Komiyama

Academic Editor

PLOS One
